# A Case of Hæmatocele, or Venous Aneurism

**Published:** 1810-10

**Authors:** J. Bradley


### *A Case of Hæmatocele, or Venous Aneurism; with Remarks, by* J. BRADLEY, M. D.

IN the year 1807, I was requested to visit a child, eleven months old, of the Rev. Mr. Matteson, at some distance from this place, who was labouring under severe symptoms of dentition. Whilst examining its gums, I perceived a tumour about the size and shape of a small pullet's egg, situated on the inferior part of the left parietal bone. On inquiry, I learnt that about six months back, whilst in its mother's arms, its head was struck against the edge or corner of a cupboard, the immediate consequence was a tumour of nearly the size it now exhibited. On examination, I perceived an evident fluctuation, but without any of that elastic feel, which is peculiar to phlegmonoid tumours; instead of which, it was looser, somewhat more flabby to the touch, and gave less resistance to the fingers, and was neither attended with pain nor discolouration. I made an incision into it with a lancet, to the extent of nearly an inch, and from the opening, a large stream of apparently venous blood issued forth. As the child had considerable fever, with great heat about its head, as well as irritation and tumefaction in its gums, I was not solicitous to suppress the hæmorrhage, till my little patient had lost as much blood as as its strength would admit. Whilst the vessel was pouring forth most copiously, I pressed my finger on the inferior margin of the tumour, and the hæmorrhage immediately stopped, but on removing it, and elevating the lower lip of the wound with a probe, I could plainly perceive the point from whence the stream proceeded, and which appeared equal to that of the median vein of an adult, when opened by a large orifice, but could not perceive the mouth of the vessel, partly as I supposed from the convexity of the cranium. There was no coagulum in the tumour, neither was the bone denuded. The distance from the line of incision to the mouth of the vessel, as nearly as I could estimate, was about three fourths of an inch. There was very little diminution of the hæmorrhage, till the patient had lost four or five ounces of blood; after which it considerably abated; but as the child seemed no ways affected by this evacuation, I suffered it to bleed about another ounce. By the time this was accomplished, the current of blood had lessened, and become little more than per stiliciduum, I therefore bound up the wound, after having applied hard compresses of linen in such a manner, as to compress the mouth of the vessel. In addition to this, I requested the parents to make a still farther pressure by the hand, for some time, and to be particularly watchful against any return of the bleeding. This careless manner of securing the hæmorrhage could only be justified by the circumstances under which I was placed, being deprived of all the means usually employed on these occasions. After imposing these injunctions, together with a request of being informed, in case of a return of the hæmorrhage, I took my leave; but in two days after I received a message, informing me, that the bleeding had returned in a most alarming degree; but before I arrived it had abated, from the sickness that had supervened. On removing the bandages, the bleeding seemed nearly stopped; but there was some coagulum in the wound.

In order to have secured the vessel by ligature, I must have divided the integuments at right angles with the first incision to have got at the vessel. To obviate this unpleasant part of the operation, I directed the hair, which was uncommonly small in quantity on that part of the head, which surrounded the wound to some distance, to be shaved, or cut close, and after having cleansed the wound from coagulum, the lips of which were brought into closer apposition, and retained by a square piece of adhesive plaister, which rather more than covered the parietes of the tumour. Over this, at the point where my finger was applied, as before mentioned, and and which effectually stopped the hæmorrhage, was laid a compress, made of about eight or ten small pieces of leather spread with adhesive plaister, an inch in length, and half an inch in breadth, laid evenly and exactly one over another, so as to form a compact solid body. Two straps of the same kind of plaister, of about six inches in length, and one and a quarter in breadth, were applied in such a manner, as to decussate exactly over this compress, and over the ends of these straps were laid others transversely, in order to secure them more effectually in their places, and to prevent them from giving way. A compress of tow and a cap were also applied over the whole. The dressings maintained their places, without affording any apparent inconvenience, for some weeks, and on the removal of which, no farther application was thought necessary.

It is a maxim, which cannot be too strongly inculcated on the minds of surgeons, never to give pain by any operation, if the same end can be equally obtained, by gentler means. Here the mouth of the vessel was obliterated by compression, between two hard bodies, as effectually, as if deligation had been resorted to. In my practice, I have met with three or four small incised wounds of the scalp, where a considerable artery has been divided, and in order to have secured which by ligature, an enlargement of the wound would have been necessary. To obviate this part of the operation, I have resorted to the compress of straps only, with the addition of the knotted bandage, having previously cut off as much hair, as would make room for the compress, which if this be done, will keep its place, a consideration of some moment. Any other kind of compress in general is not so well, on account of it being liable to escape from its place, on the bandage giving way.

Compressing the artery by the aid of the knotted bandage, I think in many instances possesses an advantage over deligation. Among timid patients, and especially females, who are horribly frightened at the name of a cutting instrument, and who can, from their usual dress of a cap, hide the whole of the dressings, I think this method of the knotted bandage is preferable. In children, however, the mode above adopted seems to me more eligible, as the dressings more effectually keep their place, and consequently are less liable to be disturbed by the little unreflecting sufferer.

But in order to illustrate the respective advantages of deligation and compression, we will suppose, for the sake of example, that a surgeon is called to a case of incised wound of the scalp, two thirds of an inch in length, accompanied with with an hæmorrhage from a divided artery. In order to secure the vessel by ligature, he is under the necessity of enlarging the wound, to double, or at least, to one third of its extent. This being effected, it follows, that in uniting the wound by the first intention, whatever number of the interrupted sutures are necessary, in the first instance, double or one third more will be required in the second; but in small incised wounds of the scalp, the use of the needle may in general be dispensed with, as has already been shewn; for the pain given by this mode of suppressing hæmorrhage is considerable; first, by enlarging the wound, secondly, by tying the artery, and thirdly, by employing the needle. Some other inconveniences attach to deligation, under the circumstances of enlarging the wound. One of these is, that ligatures sometimes rather protract the cure, as the punctures make a second wound, which is longer in healing frequently than the original one.

Another small objection against taking up the artery in these cases, after enlarging the wound, is the increased magnitude of the sore, which will in general require more time to heal than if it had been smaller.

What the practice of surgeons in general is, in cases of small incised wounds of the scalp, where a considerable artery is divided, I cannot precisely say, but I have known a few instances, where deligation of the artery, after previously enlarging the wound, has been adopted by the surgeon. It is to these practitioners, I wish to submit the above hints, being fully sensible of the necessity of the humane surgeon never losing sight of the principle of giving as little pain as possible to his patient.

This practice which I have recommended, is only applicable to such cases, as require the use of mechanical means, for the suppression of arterial hæmorrhage; for sometimes the wounded artery is so small, as to give no farther trouble, after its depletion, when the wound is united by the first intention.

Whilst describing the above case of venous aneurism, a sort of kindred affection, attended with very distressing circumstances, presented itself to my mind, which is no less than the dreadful disease, denominated by Mr. Hey, fungus hæmatodes.

Soon after this gentleman's publication was given to the world, I wrote to him, and ventured to suggest a few hints, and among the rest, were some remarks on the nature of the fungus hæmatodes. Many years before he published his work, I had given my opinion to him, as to the cause and nature nature of this truly formidable complaint, principally grounding it on some circumstances that attended the first case in his collection, and which with two others there given, happened at the time I was a pupil at the Leeds infirmary. When I first stated my conjecture, I gave some reasons why I considered it in its primary state, a varix or venous aneurism; and afterwards, I was so far convinced, that my supposition was well founded, from some of this gentleman's cases, agreeing in every particular with others published by Mr. Else, which he demonstrated by experiment to be this affection, as to induce me to hazard an opinion to Mr. Hey, though with great diffidence, respecting this new complaint.

As many of your readers may be in possession of Mr. Hey's publication, and not of the volume of the medical observations and enquiries, containing Mr. Else's paper*, a transcript of which I have thought proper to give, in order that the reader may compare his cases with some of Mr. Hey's. This paper is of considerable length, but I hope the importance of the subject will warrant its quotation.

'Every one, who, in any considerable degree, has been 'conversant with the practice of surgery, must have seen in 'different parts of the body, but most frequently in the ex'tremities, certain tumours, which having been slow in 'their progress, and appearing without any inequality, 'or discolouration on their surface (unless when the integu'ments are greatly distended) are commonly judged to be 'aneurisms, from internal causes, and when opened, are 'found to contain blood, both in a fluid and coagulated 'state. If, from the three following observations, collected 'within these two years, it should seem probable, that these 'bloody tumours are more frequently caused by the rupture 'of veins than arteries, surgeons may be hence induced to 'inquire further into this subject, and the fact being once 'well established, I apprehend the art of surgery may be 'considerably benefitted, and surgeons will not be so much 'alarmed, as they have hitherto been at these cases, but 'will attack them at their beginning, before the extravasated 'blood has had time to do much mischief:

'Case I.

'Michael Callahan, a mariner, was admitted into St. 'Thomas's Hospital, on the 30th of January, 1766, for a 'large tumour on the inside of the right arm. He said,

* Vol. iii. page 169.

'that this tumour immediately followed a contusion he had 'received, by falling with the inside of the upper part of his 'arm against the stock of an anchor, about four years before 'his admission into the Hospital: that at first, it was of a 'considerable magnitude, but that afterwards, by some 'applications, it was reduced to the size of a pigeon's egg. 'During two years it did not seem to grow any bigger, but 'afterwards, upon his being attacked with a fever in the 'West Indies, from which he narrowly escaped, it gradually 'increased; and when he came into the Hospital, was consi-'derably larger than a man's head, extending from the arm-'pit to within about two inches of the flexure of the cubit. 'The tumour was judged to contain blood, and this opinion 'was strengthened by the man's declaring that a surgeon, 'either at Portsmouth or Plymouth, had punctured it; and 'observing nothing to follow but blood, closed the wound, 'which soon healed up. On the 22d of March, 1766, he 'died. The next day the body was conveyed to the anato-'mical theatre, and while we were placing it in a proper 'position the tumour burst, and above a pint of a serous 'fluid was discharged.

'The first step taken, was to open the thorax and peri-'cardium, where all the large vessels springing from the 'basis of the heart were sound, and of their usual size. They 'were traced to the neck, but they did not appear to be de-'ceased. The right axillary artery and vein were next ex-'amined, and found to be perfect. I next opened the tu-'mour, and discovered it to be full of that coagulated mass, 'which is always contained in old tumours, formed by the 'extravasation of the blood. I then began at the axilla, to 'trace the artery, and found that it passed quite through the 'tumour without any alteration, with respect to its dimen-'sions, and without any rupture, or appearance of disease. 'The coagulated mass surrounded the artery intirely, but 'had not in the least corroded it; though it adhered closely 'in some places to its surface. I then intended to trace the 'axillary vein, and made a puncture into it, about an inch, 'before it entered the tumour, into which I thrust a probe, 'and found that it passed easily on towards the mass. 'Upon putting my finger into the tumour, I felt the naked 'extremity of the probe. I afterwards laid bare the 'deep seated branch of the basilic vein, which lies close to 'the brachial artery, just above the flexure of the cubit, and 'thrust a probe into it, which readily passed upwards; and 'upon introducing my finger again into the mass, I also 'felt the naked extremity of that probe. The parts remained in

'in this state till the day following, that the physicians and 'surgeons of the hospital might see what had been done. 'When I came the next morning, I found that the probe, 'which I had left in the axillary vein, had been pulled out, 'and I could not readily hit upon the orifice I had made for 'its introduction; I therefore made another, and introducing 'a large blow-pipe, threw in air, and observed, that the 'vein, just as it approached the tumour, was considerably 'dilated, and then opened into it. This likewise was seen 'by all the physicians and surgeons of the hospital.'

'Case 2.

'A few months ago, a woman was received into the hos'pital, for a large swelling in the calf of the leg, which was 'judged to contain blood. On opening it, fluid blood es'caped, mixed with a serous matter, and the surgeon, by 'introducing his finger, pressed out some of that mass, pro'per to these tumours. He observed on pressing his finger 'onwards, that the upper part of the fibula, for about two 'inches, was quite deficient, having been totally dissolved, 'by the extravasated blood. Upon this, it was determined 'immediately to amputate the leg. It was afterwards in'jected, by the artery, and the injection, as I myself saw, had 'passed through the posterior tibial, and fibular arteries, and 'into the anterior tibial artery. I have been informed, that 'the state of the veins was not inquired into. Though the 'rupture of the vein, in this case, was not absolutely disco'vered, yet the contents of the tumour, the dissolution of 'the bone, and the free passage of the injection through the 'principal arteries of the leg, without extravasation, seem to 'evince it.'

'Case 3.

'A young man, aged twenty-five years, of a very healthy 'habit, had a swelling in the ham. He said, that it came, 'after his endeavouring to raise a considerable weight, and 'that he felt a crack where the swelling appeared, as if some'thing had been broken. Some time in December, 1764, 'this tumour was opened, and a large quantity of fluid and 'clotted blood being discharged, it was judged to be an 'aneurism of the popliteal artery. The limb was instantly 'amputated, and immediately after the operation, carried to 'the anatomical theatre. I was myself in the country, but 'Mr. North, who then dissected for the lecturers, and to 'whose dexterity, and diligence, I am much obliged, re'ceived it, and having at that time some coarse injection

'upon the fire, while the limb was yet warm, he poured it 'into the artery, and observed it not to extravasate within 'the tumour, as he expected, but to continue its course to 'the extremity of the toes. He then looked for the largest 'internal vein, and thrusting a probe into it, found, that it 'passed readily into the tumour. He afterwards dissected 'the vein, with great caution, and discovered a rupture, just 'above a pair of valves, the edges of which were torn and 'irregular.

'Those large bloody tumours, which are seated in the calf 'of the leg, are observed frequently to follow violent fits of 'the cramp. It was the opinion of a late eminent surgeon, that 'they were produced by the Gastrocnemii muscles pressing 'the *posterior tibial* artery against the bone, and by that 'means rupturing its coats. Is it not probable, that spasms, 'blows, strains, extraordinary muscular efforts, which are 'ranked among the causes of spurious aneurism, are more 'likely to rupture the veins, than the arteries? I have seen 'several of these tumours, seated in the calf of the leg, 'opened, and in all these cases, there was a necessity for am-'putation, because the bones of the leg were found to be 'either totally dissolved in some part, or else were so much 'affected, that the fingers could be pressed quite through the 'substance of one, or the other of them, and therefore, the 'state of the artery and vein was never inquired into.

'Quere. If surgeons, by dissecting carefully these tu-'mours, whenever occasion offers, and by attending closely 'to their cause, rise and progress, may not soon be able to lay 'down very probable rules, for determining, before they are 'opened, whether they proceed from an artery, or a vein.

'The arteries sometimes become ruptured without any 'previous dilatation. I have a preparation of the aorta as-'cendens, appearing in no place dilated, which exhibits 'two ruptures; one is small, and situated about half an inch 'distance from the valves; from which a coagulum was 'formed, about the size of a large nutmeg, that was seated 'between the trunk of the aorta, and the trunk of the pulmo-'nary artery. From the white appearance of the coagulum, 'and the regularity of the edges of the rupture, it seemed to 'be of long standing. The other rupture is much larger, 'seated at the curvature, between the exit of the right and 'left carotid arteries; the edges of which are torn and irre-'gular, and formed a bloody tumour, which pressing 'against the lower part of the trachea, and the branches of 'the bronchia, destroyed the patient by suffocation, in less

'than

"than a month from its first rise, and before there was any
'appearance of external swelling.'

These remarks of Mr. Else are very interesting, and cannot be too generally known; and as it is above forty years since they were given to the public, and in a periodical work, which at the present day may not be generally read, I have thought proper to transcribe the whole of them, on account of their value, when either taken in the abstract, or considered in reference to that part of Mr. Hey's work, above alluded to.

Whoever attentively compares these cases of Mr. Else, with several of those of Mr. Hey, will be satisfied that these tumours are of the same species, as they agree, or are similar in all their essential properties. If Mr. Hey had met with these cases, in his practice, I have no doubt of his admitting them into his collection, which he denominates fungus hæmatodes. The first and sixth case of this gentleman are a counterpart to the first case of Mr. Else. The only difference seems to be, that the former has given a more minute description of his case, but the properties, essential to each, are evidently the same. The second and third case of Mr. Else, are similar to the fifth, and also to another case of Mr. Hey, given in a note, page 252, some particulars attending which he has omitted. The subject of the former of these cases was a boy, of about 14 years of age, named William Priestley; the latter was a woman of the name of Anne Hartley, aged 42 years, of a full and corpulent habit. These names I thought proper to mention, as I shall have occasion to refer to them, especially the latter, in the course of this short communication.

There are other evidences, which may be considered as collateral, but less demonstrative of this affection, being in its primary state a venous aneurism. In several places of Campinel's tumour, where the integuments were either ruptured, or very thin, the parts exhibited a bluish cast, and indeed the greatest part of the tumour assumed in some degree this appearance, and [illegible]differed from any thing of the kind I ever saw. The cracking of the integuments, with frequent hæmorrhage, rendered it not dissimilar to arterial aneurism, but the blood was not arterial, but invariably appeared venous.

Another circumstance, which seems to favour the previous supposition, as to the nature of this complaint, is the hæmorrhage of venous blood being increased, when the upper part of the thigh was compressed; also the turgidity, and enlargement of the cutaneous veins, which attended most of the cases, cases, and which were characteristic of what may be termed local and venous plethora.

The manner of Mr. Hey's accounting for the circulation in the crural artery, not being stopped by the aid of the tournequets, does not appear to me satisfactory. That two tournequets were applied, in the cases of Campinel and Ann Hartley, I can bear testimony, as well as to the effectual stoppage of the circulation, by one tournequet only, after the limb was divided; but in the latter case, the hæmorrhage was evidently diminished, before the second tournequet was secured, though I must confess, in no great degree; and even in the former case, the effusion was rather less before the stump was amputated.

In order to attempt a solution of this difficulty, it will be necessary to assume the supposition, of the case being varix. We may then conceive a very large quantity of blood, fluid as well as coagulated, contained in the sac, and the adjacent veins, surcharged and communicating with the morbid body. By forming this supposition, we may easily conceive, what a hæmorrhage will ensue, before it would be likely to stop. Mr. Hey thinks it impossible, for such a hæmorrhage to proceed from the stump; but the second fungus, made up of a conjeries of blood-vessels, together with vascular repletion, in the surrounding, or contiguous parts, seem to me sufficient to account for the great efflux of blood; and for Mr. Hey to maintain the stump incapable of supplying blood, sufficient to account for the hæmorrhage, which stopped after the limb was divided, has involved himself in an hypothesis, which cannot, in my opinion, be deemed a satisfactory explanation of this new phænomenon; and besides, it establishes a surgical, and physiological corollary, which I should not have noticed, had not the opinion of men of eminence generally given the *ton* to practice, and did not whose errors, though trifling in the abstract, lead to consequences of importance.

That part of Mr. Hey's theory, which respects the natural contractility of the capillary vessels, will be universally admitted, but in cases of amputation, most surgeons consider the tournequet as sufficient to stop the circulation, and any slight hæmorrhage, from the stump, to proceed from the part below the tournequet, and which principally consists of venous blood, regurgitating from the mouths of the divided veins. The same pressure, which stops the circulation in the arteries, as effectually prevents the venous blood from returning; but according to Mr. Hey, the tournequet is always incapable of completely stopping the circulation in the arteries; but but that a certain quantity of blood escapes, which the contractile power of the arteries is enabled to resist. This theory is certainly ingenious, but appears clogged with some difficulties, that will not be easily surmounted. How is it that this nice adaptation, in all amputations, betwixt art and nature, in every age and country, should have been so regularly and uniformly preserved. To maintain this theory, a certain quantity of blood only must be allowed to pass this instrument, and no more than the contractile power of the arteries is capable of resisting; for if the momentum of blood passing the tournequet be too large for the resisting power of the arteries, then hæmorrhage will ensue; or if a certain or given quantity of blood escape the tournequet, and there should be any diminution of contractile force in the arteries, which we have no reason to suspect ever happens, then also will hæmorrhage take place*; but if such a quantity of blood always escape the tournequet, as was apparent in the two cases, already mentioned, then any resistance in the sound capillaries would be inadequate for stopping such a torrent, and the equilibrium, which is the basis or principle of this theory, would infallibly be destroyed; for the current of blood, in both these cases, meaning those of Campinel and Hartley, were equal, or nearly so, to the unrestrained effusion of blood from the crural artery of an adult; but the pulsation in this vessel in Ann Hartley's case, previous to amputation, was stopped, therefore the hæmorrhage here must have been from another source.

The case of venous aneurism in the child above described, is no ways in favour of Mr. Hey's theory; but on the other hand, is strongly illustrative of an accumulation of blood, in the contiguous veins; for on opening the tumour, five or six times as much blood, as were apparently contained within the sac, rushed out in a powerful stream, and stopped rather suddenly on its own accord. A similar effusion of

* Observation affords few proofs of the capillaries losing their contractile power, in any local disease; for although, in gangrenous ulcerations, where this circumstance is perhaps most likely to happen, there may be a disposition to hæmorrhage, arising from a partial loss of tone in the part, yet this disposition extends no farther than the gangrene itself; for as long as there are any signs of remaining vitality, these vessels will continue to exert their contractile power in a corresponding degree, and the sound contiguous vessels, by being enabled to perform their natural functions, will be a barrier against any farther hæmorrhage. This theory is not incompatible with the all-provident wisdom displayed in the mechanism of the human frame. Next to gangrene, this supposed affection of the capillaries is perhaps most likely to occur in cancerous ulcerations; but any hæmorrhage here arises more from the virus corroding these vessels, than from any other cause.

blood blood attended the opening of the tumours, in several of Mr. Hey's cases. This circumstance, along with others, previously advanced, strongly point out the nature of the complaint, and is demonstrative of a congestion of blood in the parts adjacent to the tumour; and more especially, if we take into the account the number of arteries which were taken up at the time of amputation, in most of the above gentleman's cases where that operation was performed; and which can only be accounted for, on the principle of local plethora. It is a curious circumstance in Campinel's case, that most of the arteries secured were in the immediate neighbourhood of the tumour. In the case of Ann Hartley, there were also an uncommon number of arteries taken up. The enlargement of these vessels, it is evident, was not owing to any pre-existing inflammation in the morbid parts, or their vicinity, as is generally the case, but from distension in consequence of an increased accumulation of blood in the adjoining parts. As to the veins, we know that they are capable of very great distension from an obstruction in the circulation, as we frequently observe in the lower extremities, in consequence of pregnancy. In these cases, alarming hæmorrhages sometimes burst forth from a cutaneous vein, which, when greatly varicose, will pour out a stream that will not stop of its own accord, till the patient has in general lost an uncommon quantity of blood. These veins, when once distended, do not always regain their former dimensions; for I have known several instances of their continuing enlarged for several (even twenty) years subsequent to the patient having children, when suddenly one of them has burst, and poured forth a large stream corresponding with the diameter of the vessel. The rupture is so sudden, and the current so large, that in a short time the patient has sustained a very profuse hæmorrhage; and she or the bye-standers will often tell the surgeon, that had it not been for such an application or remedy, meaning the last that was adopted, she would certainly have bled to death before his arrival; but in truth the bleeding here, after some time, is generally disposed to stop of its own accord; but it must be confessed, however, that the hæmorrhage always has a very alarming appearance.

If the tumour of Master Mattison had contained any coagulum, I believe Mr. Hey would have classed it among that description of tumours which he designates by the name of Fungus hæmatodes. Now, as it is clear this is a case of venous aneurism, and it is equally true that Mr. Else has shewn, that in one of his cases of venous aneurism, the tumour contained a large firm mass of coagulated blood, it follows, and is is more than probable, that the venous, like the arterial aneurism, begins with fluid blood, and passes through the different degrees of coagulum, till it arrives to a state of as great firmness as it is capable of. It hence also follows, that the primary stage of fungus hæmatodes is nothing else than venous aneurism; and as the case of the child has already been demonstrated to be this latter affection, it consequently ought to be admitted into the class of fungus hæmatodes.

The coagulum, in the case of William Priestley and Ann Hartley, was not proportionally large, as in Campinet's case, and less consolidated. If I may so express myself, the blood seemed nearly half formed into coagulum. If the different degrees of density of these tumours be admitted, it follows that Mr. Hey's criteria, or directions for ascertaining this complaint, are somewhat equivocal; for, according to which, if this case of the child be classed among these tumours, who could determine the nature of the case? On the other hand, provided the surgeon had never seen such a case as Campinet's, nor any similar to it, in point of even and uniform density of the coagulum, how could he ascertain the nature of it according to these directions.

Hence we may infer, that these tumours exhibit different degrees of density at their respective stages, and in some respects are a good deal alike arterial aneurism. The blood in both will continue some time in a fluid state, most probably, till the coats of the vessel lose their tone, after which coagulum forms or accumulates by degrees: and as the tumour advances in size, it increases in proportion to its contents, till at length it cracks in one or more places. From these small fissures, or orifices, different or even fatal hæmorrhages may possibly ensue. The coagulum in Campinet's tumour is well described by Mr. Hey, and certainly bled on being broken, and which led him to suppose it was organized, an idea which seems to have been strongly impressed on his mind. In my correspondence with this gentleman I ventured to hint, that coagulum, as long as it continued to have connection with the source that gave rise to it, and was constantly soaked or macerated in warm fluid blood, which was constantly circulating within the sac, would bleed on being broken; and on this principle I endeavoured to account for the cause of the apparent vascularity of this substance.

The coagulum in Campinet's tumour did not appear to me to adhere firmly to the sac; but, I thought, might have been separated from it by the hand, without much force; but this did not seem to be exactly the case with the second fungus, which appeared to adhere more firmly, and was of rather a more gelatinous consistence.

What relation these two fungi bore to each other, I cannot say; but I have long considered the first, as being nothing else than firm coagulum, and the second, a real fungus growing out of a diseased sac. The case of Richard Finney, in Mr. Hey's collection, strongly favours this conjecture, for here we see the coagulum destroyed by the seton; but the second fungus, that arose from what we conceive to be the diseased state of the sac, could not be destroyed by the same means.

There is another circumstance peculiar to this affection, and which renders it somewhat analogous to arterial aneurism. Whenever this tumour lies contiguous to a bone, it will incontestably destroy it; but this destructive principle I suspect, is more active in venous, than arterial aneurism. In the case of Ann Hartley, about an inch and a half of the fibula was destroyed, and an osseous substance, mixed with coagulum, either lying in the vacuity or very near it. In one of Mr. Else's cases, also, a part of the bone was wanting. I mentioned the circumstance of part of the fibula being dissolved, in Hartley's case, to Mr Hey, after the limb was examined (for she was not this gentleman's patient) and by not availing himself of this hint, he subsequently lost the opportunity of inspecting the state of the bones in two cases. His words are, " a few years ago I amputated the arm of a middle-aged man below the elbow, who had a tumour exactly similar to the last described; but the state of the bone was not examined, nor did I examine it in the case of Mr Ward, having seen no affection of the bone from it at that time." Hence, if the contents of this tumour be capable of making such ravages among the bones, we cease to wonder at the morbid appearance, in the soft contiguous parts, and at the sac being incapable of sending forth good granulations.

If the above theory be admitted, it follows that the primary state of fungus hæmatodes is venous aneurism, and which may be divided into two stages, for practical purposes. The first is, that which consists of fluid blood entirely, and where the sac is in a sound state. Here a simple division of the tumour, and securing the vessel will probably be sufficient, as was practised in the case already described. The second stage is, where the contents of the tumour consist of coagulum. Here the sac may be conceived to be in a morbid state, and capable of throwing forth a fungus, which properly constitutes the disease designated by the term above-mentioned.

Wherefore, if this tumour containing fluid, as well as coagulated blood, be venous aneurism, as there is reason to believe, believe, it is obvious that what are denominated the two stages of the same complaint, constitute two distinct diseases. This is exemplified in case 1st of Mr. Hey's collection, where we see all the coagulum removed, and only a small part of the sac remaining, from which grew the substance, which ought to be considered only as a fungus. One particular relating to the case of William Priestley, I had forgot to mention in its proper place, and which was the quantity of blood which he lost during the operation, and which was greater than what usually occurs in similar cases of amputation, and the consequent sicknes that ensued. It must be remembered here, that the limb was amputated above the knee, and the disease was in the calf of the leg.

The following case will prove the inefficiency of two tournequets, for suppressing hæmorrhage, from the end of the stump, in amputating one of the lower extremities, for an affection different from the fungus hæmatodes.

On the 12th of May last (1810), I was requested to be present at the amputation of the leg of a man, of the name of John Beaumont, aged 34 years, of a very cachochymic and reduced habit of body. The operation was in consequence of a white-swelling of the knee, of some years standing, and which was of a very large size. He had employed a variety of means, both internally and externally, and particularly blisters, which were continued for several months successively, and renewed, or repeated, as often as the part cicatrized. The patient was in an emaciated state, had a quick low pulse, at 106, a slightly furred tongue, and night sweats, but without symptoms of any other local, or general affection.

After securing the tournequet effectually on the upper part of the thigh, I was much struck at the quantity of dark-coloured, or venous-like blood, that followed the division of the integuments, and which continued to increase, as the operator prosecuted his incision through the muscles. The hæmorrhage, by the time the muscles were divided down to the bone, was so profuse, that it was judged necessary to apply a second tournequet to the middle of the thigh, and which, together with the first, though tight, did not seem to have any effect in checking the effusion of blood; for whilst the bone was sawing through, a considerable quantity still continued to flow uniformly from both the superior, and inferior portions of the divided limb. There was no apparent bleeding from the crural, or any other arteries of the thigh. As soon as the bone was sawed through, the hæmorrhage considerably abated, and before half the interval transpired, that was spent in taking up three or four small arteries, arteries, it was reduced to little more than a general oozing, from the end of the stump. In about fifteen or twenty seconds, as nearly as I can estimate, after the crural artery was tied, a large stream issued forth, from the mouth of the great vein, which was soon stopped, but whether, by a farther tightening of the tournequet, or what is more probable, from the pressure I made with my finger, on one side of the mouth of the vessel, so as to afford the operator, room for securing it with an armed needle, I cannot say. All the smaller arteries, previously to their being tied, on slackening the tournequets, threw out arterial blood, of quite a different colour from the hæmorrhage already mentioned, and which was as often suppressed, as the tournequet was again tightened, exactly as we see in amputations in general. After all the arteries were taken up, and the circulation set at liberty, the stump did not seem disposed to bleed any more than what is customary in such cases, notwithstanding the application of a moderate artificial heat for some time, to the end of the stump, in order to relax the mouths of the divided vessels. The muscles appeared darker coloured than usual, and that part of the thigh, which was operated upon, seemed to me not quite so small as might have been expected, from the emaciated state of the body, and the size of the swelling. The cutaneous veins of the knee were enlarged, but no more than we frequently see, in affections of this nature. The knee, on examination, displayed those morbid appearances, which we usually meet with, in scrophulous swellings of the joints; and the ancle joint, also exhibited signs of a beginning state of a similar disease; and the whole leg, especially the lower part of it, was somewhat swelled and œdematous.

The man was sick after the operation, yet for the first two days went on tolerably well, though his pulse evidently portended debility, as they had not acquired any force, as is usually the case at this period; but at the beginning of the fourth day, he was seized with vomiting, and constipation of the bowels, which yielded not to any means that were prescribed. His pulse were nearly at the same number they were, previous to the operation, but evidently was sinking. He complained of pain, both in the epigastric, and hypochondriac regions, and the fluid that he vomited, had a greenish, and a somewhat yellowish cast, and was sweet to the taste of the patient; but as his disorder advanced, it assumed a bloody appearance. He had but little thirst, yet his tongue continued rather more furred, than before the operation, and his stump looked well. On the fifth day, however, his pulse sunk, and were at 112: his countenance became more ghastly, respiration short, and somewhat an helose, his eyes glistened, delirium and cold clammy sweats succeeded ceeded, and death, the sixth day after the operation, closed the scene.

Here the integuments and muscles, on their division, displayed the same dark colour as the consequent flow of blood; and the greatest current of the hæmorrhage was at the precise juncture of dividing the muscles, and which seemed to abate insensibly till the limb was amputated, without the influence of any means, that were employed for stopping it. The stump, after the hæmorrhage ceased, had rather a fresher and more oxegenized aspect.

In Priestley's and Hartley's cases, who both lost an unusual quantity of blood, especially the latter, the same phœnomena were observable, as to the colour of this fluid, and the hæmorrhage, which burst forth occasionally from Campinet's tumour, before the first amputation, displayed a similar cast.

Of the quantity of blood, lost in this case of amputation, I can form no correct idea. I think it could be no less than from thirty to forty ounces, and which greatly diminished the force of the pulse, at the time of the operation; and though they acquired some small re-accession of strength, in consequence of the symptomatic fever, for the first two or three days, yet, as soon as this subsided, the real state of the system unfolded itself, and the patient sunk more from extreme debility, than from any other cause.

I do not know of any case of amputation on record, for a similar affection, attended with such phœnomena as the above; for though Mr. Hey mentions his having seen the circumstance of two tournequets being insufficient to stop the circulation in the artery, more than once, he only alludes to the cases of Campinet and Hartley, both of which were of the fungus hæmatodes. The most obvious way here, in my opinion, of accounting for this extraordinary phœnomenon, is on the principle of local plethora, as above mentioned, or by supposing the veins of the limb surcharged with blood stagnating from a languid circulation, or from some other cause; for the hæmorrhage seemed to flow from the under, as well as the upper part of the limb, when divided; and this circumstance will account for a good part of the blood, that was lost; for as soon as the limb was amputated, the hæmorrhage from the stump could be more clearly ascertained, and which was very considerably lessened, as before stated.

The pressure on the thigh was very great; for on the first appearance of an increased effusion of blood, the first tournequet was further tightened, insomuch, as to give considerable pain; the second also was screwed to a similar degree.

This

This case, by being different from those of the fungus hæmatodes affords apparently other data, for accounting for the singular hæmorrhage, that attended it; and which has never before been found to occur in amputation, that I know of, except in Mr. Hey's cases, from the supposed morbid state of the capillaries; but here there can be no ground for suspecting any defect in these vessels. On the contrary, this case militates strongly against Mr. Hey's theory. For to say that the efflux of blood was occasioned from a disease in the capillary arteries, is to assert a solecism, in the face of facts and probability; for the blood, that flowed from the divided parts, seemed not only general, but venous and dark-coloured; and the part operated upon, had no appearance of disease externally; and internally, every part of the limb, bore a similar aspect. Of all the phœnomena attendant on this case, none perhaps is more difficult to account for, than the large stream of blood, that burst out from the mouth of the crural vein. If we admit the principle of local plethora, as above mentioned, we then may conceive this vessel, surcharged with blood, especially after the tournequet was tightened, partly from the smaller veins emptying themselves into it, and which this supply would every moment more and more distend, till at length the valves became ruptured, or gave way, and consequently the regurgitating blood would rush out, with considerable force, as was actually the case, from the enlarged or distended state of the vein.

The above remarks on fungus hæmatodes, comprize nearly the substance of my private communication to Mr. Hey, on this complaint, and had I not met with two such cases, as are above detailed, the observations accompanying them would never have met the public eye. Wherever I have differed from this gentleman in opinion, as to either theory, or matter of fact, I have in the one instance assigned my reasons, such as they are; and as to facts, I have described them, as they appeared at least to me, with a proper and high respect for Mr. Hey's abilities, and a becoming diffidence of my own. In this gentleman's polite and friendly answer to my remarks, he rightly observed, that some of his cases could not be dependent on the cause which I had assigned, and particularly mentioned the case of Mrs. Storr; but in reply, I must beg leave to say, that I endeavoured to prove no more than that several of his cases were similar to those of Mr. Else, which he demonstrated *by experiment* to be venous aneurism; therefore, the obvious inference must be such, as I have already deduced.

*Huddersfield, July* 10, 1810.

