# Errata

**Published:** 1810-10

**Authors:** 


### ERRATA.

In the Paragraph in Italic at the beginning of the Number, line 7, for "*in the* respect," read "in *this* respect."

