# Errata

**Published:** 1810-10

**Authors:** 


					Jn the running head of Dr. J. Bradley's Case, for " J. Bradley," read " Dn
? J. Bradlty." ? .

				

